# Prejudice and Health Anxiety about Radiation Exposure from Second-Generation Atomic Bomb Survivors: Results from a Qualitative Interview Study

**DOI:** 10.3389/fpsyg.2017.01462

**Published:** 2017-08-30

**Authors:** Yuka Kamite

**Affiliations:** Department of Psychology, Graduate School of Education, Hiroshima University Higashihiroshima, Japan

**Keywords:** second-generation atomic bomb survivors, prejudice and discrimination, intergenerational transmission of trauma, radiation exposure, health anxiety

## Abstract

The effect of atomic bomb radiation exposure on the survivors and their children has been a worrisome problem since soon after the 1945 Hiroshima and Nagasaki bombings. Researchers have examined physical and genetic effects; however, no research has focused on second-generation survivors’ (SGS) psychological effects. Consequently, this study shed light on the SGS’ experience of discrimination and prejudice and their anxiety concerning the genetic effects of radiation exposure. This study utilized semi-structured interviews with 14 SGS (10 women, mean age = 56 ± 6.25 years, range = 46–68 years). Data were analyzed using a modified version of the grounded theory approach. Three categories were extracted: low awareness as an SGS, no health anxiety regarding the effect of radiation, and health anxiety regarding the effect of radiation. The results did not reveal that SGS who grew up in the bombed areas experienced discrimination or prejudice. They had little health anxiety from childhood to adolescence. In this study, some of the SGS developed health anxiety about their third-generation children, but only among female participants. Perhaps the transgenerational transmission of anxiety concerning the genetic effects of radiation exposure causes stress, particularly among women with children. However, a change was seen in adulthood health anxiety regarding the effects of radiation, suggesting the possibility that changes in the psychological experiences of SGS can be observed throughout their lifetimes and that their own health status, and that of their children, the third-generation survivors, affects their health anxiety regarding radiation.

## Introduction

Seventy-two years have passed since the first atomic bombs were dropped on Hiroshima and Nagasaki in August 1945. It is estimated that 210,000 people lost their lives in these bombings. Furthermore, the health of the victims who subsequently survived was threatened by the damage caused by radiation. Several medical studies have addressed these types of atomic bomb injuries such as cancer risk (Shimizu et al., 1990), leukemia morbidity (Preston et al., 1994), and microcephaly due to in-utero radiation exposure (Miller and Blot, 1972).

Research published thus far regarding the psychiatric effects of the atomic bombs has only addressed people’s reactions initially after the bomb (Okumura and Hikita, 1949), 8 years after the bomb (Konuma et al., 1953), and 11 years after the bomb (Nishikawa and Tuiki, 1961). However, the dearth of studies from a psychiatry and psychology standpoint has been noted (Ohta et al., 2000; Honda et al., 2002). Among them, R. J. Lifton’s work is notable for understanding the psychodynamic aspects of atomic bomb survivors. Lifton (1967) conducted interviews in Hiroshima, and published the results in *Death in Life*. Lifton identified “death imprint,” “survivor guilt,” and “struggle for meaning” as psychological characteristics of atomic bomb survivors. Furthermore, Lifton said that the radiation effects were to be long lasting; in some cases there would be physical terror that would continue throughout individuals’ lives. This would not be limited to survivors alone, but would extend to their descendants. For example, survivors worried about the genetic impact of radiation on their children. Therefore, shedding light on what the children experienced provides an important reference point when examining the long-term effects of the atomic bomb.

Moreover, in the latter half of the 1990s, the concept of PTSD became widely known in Japan. Attention was drawn to the PTSD of atomic bomb survivors, and large-scale systematic surveys were conducted. The Ministry of Health, Labor and Welfare (2001) showed correlations between surviving the atomic bomb and reduced current self-rated health status and an increase in history of disease. It is very hard to imagine that the varying health problems are due to direct radiation effects at the time the bombs were dropped. Rather, there is a high probability that the problems are due to anxiety triggered by the experience. It can be presumed that anxiety about possibly having been exposed to harmful radiation and social prejudice are important elements. Research conducted in recent years regarding PTSD among survivors suggests that health anxiety about the effects of radiation will continuously affect survivors.

To detect physical and genetic effects, since the latter half of the 1940s, the Radiation Effect Foundation has performed follow-up surveys with Hiroshima and Nagasaki survivors and SGSs to address the transgenerational effects of radiation exposure. Even though there have been many other studies, at present, there is no empirical support for clear genetic effects on SGS (National Research Council, 2006; Grant et al., 2015). Research has also failed to address SGS’ psychological outcomes. However, the victims experienced one of the worst calamities in history. Therefore, the effects on subsequent generations has extremely important historical significance.

The only quantitative, empirical, and investigative studies to date were conducted by Tomoike (2007) and Ben-Ezra et al. (2012). The only qualitative study was conducted by Sawada (2011). Since the 1960s, research has thoroughly examined the Holocaust, which is similar regarding its historical trauma due to war. By the latter half of the 1990s, over 400 book-length research studies had already been published regarding the Holocaust (Kellermann, 1999). By comparison, it must be said that research on SGS of the atomic bombs is delayed.

Tatara (1998) was the first researcher to write about the psychological effects of SGS. The findings were that bomb survivors who were parents were afraid that their children would suffer social prejudice; therefore, they hid the fact that they were survivors from the people around them, which seemed to torment them. However, Tatara only looked at survivors and their anxiety about the second-generation, not examining the subjective psychological experiences of the SGS themselves.

Additionally, Tomoike (2007) conducted a pilot, qualitative survey of health anxiety among second and third-generation Nagasaki survivors. This study addressed genetic effects of the atomic bomb and attempted to elucidate health tendencies (e.g., illness and anxiety) among survivors. Tomoike examined whether survivors felt that there would surely be effects on their descendants, and if they felt that they would not be able to tell whether there were genetic effects.

Furthermore, in their study of fear of radiation exposure and PTSD symptoms after the March 2011 Fukushima nuclear incident, Ben-Ezra et al. (2012) compared their participants to a control group of third-generation atomic bomb survivors living in Hiroshima and Nagasaki. The conclusion was that grandchildren of Japanese people living in Hiroshima and Nagasaki showed more fear of radiation exposure and higher levels of PTSD symptoms. This quantitative study suggests the possibility that daily awareness of the genetic effects exerted an influence on the health anxiety of third-generation survivors and that when an event, such as a nuclear incident, increased the threat of radiation exposure, anxiety levels over radiation differed depending on whether a person was a descendant of an atomic bomb survivor or not.

Sawada (2011) conducted the first qualitative research studying the descendants of atomic bomb survivors. She conducted interviews with three generations of three families—atom bomb survivors, SGS, and third-generation survivors—which was modeled on research about three generations of Holocaust survivors (Bar-On, 1995). The results suggest that SGS do not hear very much about the atomic bomb experience from their parents, that their awareness of themselves as “SGS” has become attenuated, and that they have experienced almost no discrimination or prejudice. Sawada also suggested that a portion of the second-generation has inherited health anxiety. These results indicate the need for a more detailed investigation of the psychological problems experienced by SGS—specifically, how health anxiety arises.

Consequently, this study examined the long-term psychological effects of SGS including anxiety about the genetic effects of radiation exposure and experiences of discrimination and prejudice.

## Materials and Methods

### Participants

The sample of this qualitative study was initially recruited from participants of events for atomic bomb survivors, personal contacts, and, eventually, snowball sampling. Participants comprised 14 SGS (10 women, mean age = 56 ± 6.25 years, range = 46–68 years). Participants had either a mother, father, or both parents survive the bombings. Thirteen SGSs grew up in Hiroshima or Nagasaki; one grew up in a non-bombed area. At the time of the interview, 10 SGSs were married, two were divorced, and two were single. Twelve had children and two did not. Concerning survivor parents, 14 were inside the cities when they were bombed (directly bombed), 4 entered the cities within a few days after the bombing (indirectly bombed), and 1 was a nurse of bombing victims. SGS ranged in age from 6 months to 38 years at the time of the attack.

### Procedure

All eligible individuals were given or sent an invitation letter, which was followed by a phone call or an e-mail from an investigator who explained the general purpose of the research. The investigator conducted all interviews at a location chosen by the participant such as their home, the investigator’s university, or civic centers. The interviews were conducted in Japanese and lasted between 2 and 3 h. Interview data were digitally recorded on an audio recorder after obtaining the participant’s consent, and transcribed verbatim.

### Ethics Statement

Prior to the interviews, all participants were assured that their anonymity would be maintained. I informed each participant about the purpose of the study both verbally and in writing. I also informed them that their participation in the study was voluntary and that refusal to participate would not disadvantage them. Then, they completed a demographic questionnaire and provided signed informed consent. This study was approved by the Ethics Committee of the Graduate School of Education, Hiroshima University and was conducted in accordance with the Declaration of Helsinki.

### Interviews

Data were collected under a semi-structured interview format. The investigator used open questions in a conversational style to facilitate participants’ talking about diverse topics: (1) parents’ experiences of the bombing and life experiences after the war up to the present, (2) experience of prejudice as an SGS, and (3) recognition of the hereditary influence of radiation.

### Data Analysis

Data were analyzed using a modified version of the grounded theory approach (Kinoshita, 2003), which was developed by adopting its theoretical and content properties (Glaser and Strauss, 1967). A detailed data analysis was conducted accordance with Kinoshita (2003), employing the following procedure. For the analysis, we started with data from survey respondents whose interviews were completed first. Based on the analyzed data, we extracted concrete examples with close relevance to the experience of prejudice and discrimination as a SGS and anxiety concerning the genetic effects of radiation exposure; then, these concepts were shaped. We compiled the concept names, definitions, and concrete examples on an analysis worksheet. In the second and subsequent examples, we generated new concepts while comparing and examining similarities and opposite examples. Next, we carefully examined the relationships with the concept questions and created categories out of multiple concepts. With the cooperation of two graduate students majoring in clinical psychology with experience in qualitative research and one university instructor, we carefully checked the suitability of the concepts and categories for the analysis process. We concluded the interviews at the stage where the extraction of concrete examples and the work of concept creation had reached the saturation point. Then, we created a results diagram from the interrelationships among the final concepts and categories. Subsequently, with the cooperation of two clinical psychologists, we investigated the suitability of the final concepts, categories, and the results diagram.

## Results

**FIGURE 1:**
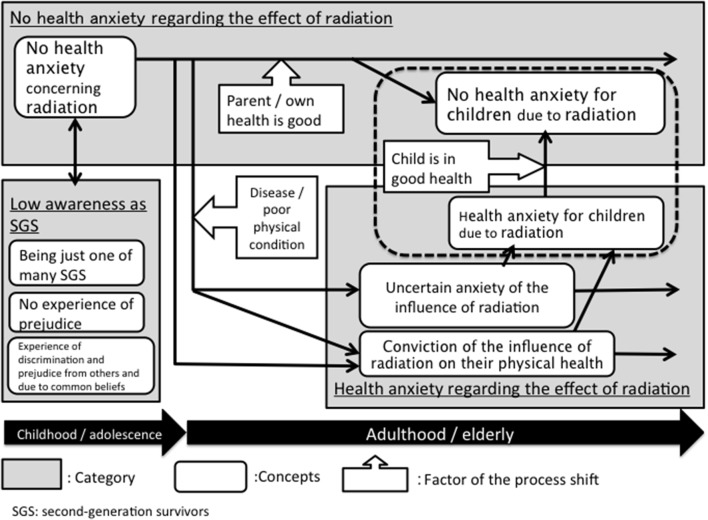
Second-generation atomic bomb survivors’ health anxiety and awareness.

Three categories were extracted: low awareness as an SGS, no health anxiety regarding the effect of radiation, and health anxiety regarding the effect of radiation. The interrelationships among the categories and the concepts that make up them are shown in the results diagram **Figure 1**. Below, I will explain about each of the three categories.

### Low Awareness as an SGS (Category 1)

This category consisted of three concepts: being just one of many SGS, no experience of prejudice, and experience of discrimination and prejudice from others and due to common beliefs. The following section defines each concept and provides concrete examples in the survey respondents’ own words.

#### Being Just One of Many SGS

Most participants grew up in the bombed areas; therefore, they did not have a special meaning about being a SGS from their childhood to early adulthood. In particular, for the SGS who grew up near the bombed areas, seeing the keloid scars left behind on their parents’ bodies and on the bodies of the adults in their neighborhoods and seeing the barracks-like buildings during the restoration period were common. As the following two cases show, most SGS felt that they were just one of the many SGS:

*Everyone around me was an atom bomb survivor; son all I knew was that pretty much everyone out there were bomb survivors*.

People weren’t hiding anything. In daily life, it was not a big thing that there were elderly women who had those keloid scars. The way we learned that they had them because of the atom bomb was just matter-of-fact information.

#### No Experience of Prejudice

None of the survey respondents had experienced discrimination because they were SGS. Many respondents spoke about not having been discriminated against or receiving prejudice:

I, myself, did not experience this type of discrimination, and my mother never said that she had been discriminated against.

#### Experience of Discrimination and Prejudice from Others and Due to Common Beliefs

No direct experiences of discrimination were mentioned in this study; however, the experiences of other SGS were spoken of in general terms:

Sure, like being told ‘it’s catching.’ That’s not bullying; but, there were lots of people who were made to feel bad. We were told about things like that in Peace Education class; but, I, myself, did not experience it.

Additionally, there was one participant who grew up in a different region than the bombed areas. Even as she neared adulthood, her mother and grandmother still hid the fact that her mother was a survivor because they were afraid of prejudice and discrimination:

My grandmother told my mother that, particularly for females who would be bearing children, that this would make it hard to get married; so, she was absolutely not to tell anyone about being an atom bomb survivor, and she kept quiet about it all that time.

Since her mother kept it a secret that she was a survivor, Participant 10 did not develop consciousness that she herself was an SGS until after she became an adult. From this example, it is conceivable that prejudice and discrimination were more pronounced in the non-bombed areas than in the bombed areas.

### No Health Anxiety Regarding the Effect of Radiation (Category 2)

This category includes cases where SGS do not have anxiety about the effect of radiation exposure due to the bomb, neither for themselves nor for their children (i.e., the third generation). This category consisted of two concepts: no health anxiety concerning radiation and no health anxiety for children due to radiation.

#### No Health Anxiety Concerning Radiation

None of the survey respondents were anxious about their health due to the fact that they were SGS when they were children or adolescents. However, a variety of changes in health anxiety were observed in the second-generation starting in adulthood. Some of the SGS respondents indicated that they did not have health anxiety about the effects of radiation exposure prior to the interview. The points in common were that the SGS respondents, who until that time did not have health anxiety, had atomic bomb survivor parents who were in comparatively good health, and they themselves had not experienced any major physical disorders up to that time. Additionally, one participant said that she did not feel anxious about her health until she was diagnosed with cancer as an adult:

Even though I went to the health checkups for second-generation survivors, somehow, I didn’t feel that it had anything to do with me. When I read the sentence about health checkups for second-generation survivors, of course it referred to me as well, and I wasn’t an outsider in a sense; but, it was somebody else’s business.

She had received periodic health checkups; however, she had not felt health anxiety until she was diagnosed with cancer as an adult. She mentioned that after being diagnosed with cancer, she began thinking about the effects of radiation exposure on her child as well. Changes to her health anxiety levels could be seen throughout her life due to her health condition and the presence of a child.

#### No Health Anxiety for Children Due to Radiation

This concept indicates the state of not being concerned about the genetic effects of radiation exposure on one’s children. If the second-generation themselves did not have health anxiety, no health anxiety was observed in their third-generation children. In these cases, as recounted below, they had not experienced anxiety in the first place; therefore, their responses were extremely simple:

I don’t really remember; but, I don’t think I was all that concerned. (Perhaps I was concerned) The normal amount. Both of them were born healthy.

Furthermore, as recounted below, in some cases anxiety was felt when the first child was born; however, not when the second child was born:

(Concerning when I was pregnant with my first child) When you are pregnant, you feel a certain amount of anxiety, and as far as health was concerned, if we weren’t atom bomb survivors, everything would be clear; but, there was just a tiny bit of darkness at that time. It gradually went away; but, part of it might remain somewhere. I don’t know; but, I’m not hiding it. (About how the anxiety went away after the eldest child, a daughter, was born healthy, and about the birth of the second child, a son) More than any anxiety, there was pressure to produce a son.

In this example, the health anxiety that was felt when the daughter was born disappeared when she was born healthy. When the second child was born, the concern transferred to the pressure that was felt regarding producing a son and heir. This shows that, as with health anxiety of SGS themselves, health anxiety for their children can appear and disappear according to the health status of the children and their own health status.

### Health Anxiety Regarding the Effect of Radiation (Category 3)

This category includes statements made concerning health anxiety about whether they or their children had been affected by exposure to radiation from the atomic bomb. This category consisted of three concepts: uncertain anxiety of the influence of radiation, conviction of the influence of radiation on their physical health, and health anxiety for children due to radiation.

#### Uncertain Anxiety of the Influence of Radiation

Even second-generation respondents who did not feel anxious about their health as children or adolescents said that they began to have health anxiety over the genetic effects of radiation exposure after they became adults. For example, when they thought about their health, they experienced anxiety in the form of uncertainty about whether they would get sick due to the effect of the atomic bomb radiation, or thought that the health issues they were currently experiencing were probably due to radiation effects. Many SGS expressed that they did not feel anxious all the time. When they felt unwell, the anxiety increased and receded in short order; it would momentarily cross their minds. The following is a concrete example of this:

It hits me when I have a checkup and my white blood cell count is high, even though I am not sick. It hits me a little when I give blood. The thought comes up, could this be because I am a second-generation survivor? It is really only for an instant. Something like, ‘it must be because I am a second-generation survivor.’ When I hear the stories, I was told that there is no damage to health of members of the second-generation; but, there are a lot of times when I think that there must be.

It can be seen from this account that many SGS encounter information that says SGS are not affected by radiation. However, this indicates that possessing this knowledge does not necessarily relieve anxiety directly. Specifically, after having experienced physical symptoms that have a high correlation with radiation damage, such as an elevated white blood cell count or being diagnosed with cancer, SGS became more likely to experience anxiety. Furthermore, the SGS who develop health anxiety were influenced not only by their own illnesses, but also family members’ illnesses.

#### Conviction of the Influence of Radiation on Their Physical Health

We observed that some SGS expressed the conviction that they suffered genetic consequences due to radiation exposure. This was observed as an awareness that their cancer was due to genetic effects of radiation exposure, or the awareness that they were destined to develop cancer in the future. The following is a concrete example of this:

I developed a polyp on my throat about 10 years ago, and when I got it examined, I discovered that I had a lot of goitrous tumors. When I was told that I had hypothyroidism, I thought that this was the effect of being a SGS. My father also had hypothyroidism, and he had to take Levothyroxine. So, I thought, now it’s my turn.

In contrast to the health uncertainty anxiety mentioned earlier, this indicates the awareness of being affected genetically by radiation exposure more clearly. There were two types of respondents who were certain they were affected genetically. They were ones who had themselves developed cancer, and ones who felt certain they would even though they had no health problems. Furthermore, we observed two types of health statuses: those who were well and those who had ongoing physical conditions. We were unable to find a simple pattern in the respondents’ or parents’ health status as a cause of the certainty of genetic effects. We believe that it is possible that a variety of other causes are involved.

#### Health Anxiety for Children Due to Radiation

The concepts outlined thus far concern health anxiety of the SGSs themselves. This concept includes anxiety about the genetic effects on their children, the third generation. Some of the SGS who had children expressed anxiety that their children might also be genetically affected by the radiation. However, this result was only seen in female participants:

(After being diagnosed with cancer myself) I started thinking things like, what happens if I pass it on to my daughter, or what will I do if something happens? … Even though I thought that there was almost no effect on the second-generation, it comes up a long time later; after all, genes are passed on, and in my case both my parents were atomic bomb survivors: both my mother and my father. Therefore, my children have those factors, right? They will be the third generation; but, you can’t say absolutely that there is no effect.

In the same way as there was health uncertainty anxiety about themselves, they spoke of their anxiety that it was not certain that there would be no radiation effects on their children. Moreover, this anxiety was activated when the SGSs themselves became ill, when their children became ill, and when their children married.

## Discussion

This study qualitatively assessed the psychological aspects of SGS concerning the experience of discrimination and prejudice and anxiety about the genetic effects of radiation exposure. We discuss these themes below in the context of the attenuation of awareness of oneself as an SGS and from the standpoint of health anxiety about radiation exposure.

### The Context of the Attenuation of Awareness of Being an SGS

We have noted that the survey respondents in this study had the common experience of low *awareness* of being SGS during the time between childhood and early adolescence. These results are like those of Sawada (2011). One factor in this is that all participants in this study except one were born and raised in Hiroshima or Nagasaki, the cities that were bombed. We believe that because of this, being an SGS was not perceived as special.

Regarding this point, several studies have examined second-generation Holocaust survivors and the psychological impact of historical trauma (also due to World War II) on the next generation. Recent studies on the intergenerational transmission of trauma in non-clinical samples found no evidence that Holocaust survivors’ traumatic experiences affected their offspring’s adjustment (e.g., Sagi-Schwartz et al., 2003, 2008; van IJzendoorn et al., 2003; Levav et al., 2007; Iliceto et al., 2011). However, secondary traumatization occurred in studies on clinical populations of offspring who suffered from vulnerability to PTSD (Yehuda et al., 1998, 2002), difficulties in coping with stressful experiences (Baider et al., 2000), and poor attachment styles (Bar-On et al., 1998).

Using non-clinical atomic bomb SGS, the current study found that the survey respondents had the common experience of attenuated *awareness* of being SGSs during the time between childhood and early adolescence. This finding, as well as no evidence of Holocaust survivors’ trauma on their offspring’s adjustment in non-clinical samples, suggests little or no effect of atomic bomb survivors’ trauma on their children and adolescents’ adjustment in non-clinical samples.

Furthermore, many studies have addressed survivors who immigrated to other countries. Braga et al. (2012), in their study of transgenerational transmission of trauma among second-generation Holocaust survivors who immigrated to Brazil, noted the challenges of adapting to a new country. In this study, the SGS raised in the bombed areas did not face the problem of adapting to a region. On the contrary, it is conceivable that the sense of togetherness as a community with a shared experience suppressed prejudice and discrimination. In this way, the experience of growing up in the bombed areas made it unlikely for them to have negative experiences. We believe that from childhood to adolescence being an SGS held no special meaning for them.

However, several survey respondents indicated that they knew of the existence of prejudice and discrimination from information at school, in the media, or from the experiences of acquaintances. Because they only came in contact with the information indirectly, this did not generate any emotional responses such as anxiety or anger. Similarly, in Sawada’s (2011) survey, the second-generation did not mention experiencing direct discrimination or prejudice. However, there was one female participant in this study who was raised in a non-bombed area. She recounted that her grandmother and her mother were afraid of the negative impact on her opportunities for marriage, and they hid the fact that she was an SGS. The possibility exists that prejudice and discrimination against atomic bomb survivors and SGSs was more likely to occur in regions that had not been bombed. Furthermore, this way of maintaining relationships by the mother keeping her survivor status secret from the family and neighbors, as demonstrated in this case, has also been reported similarly in studies of intergenerational transmission of trauma relating to the Holocaust (Danieli, 1998). This study did not examine what kind of communication is used within families to “pass down” the traumatic story. However, the context of the attenuation of the awareness of SGS from childhood to adolescence makes it conceivable that they were not told very much about the atomic bombing experience. In the future, a study from the standpoint of intergenerational transmission of trauma of how atomic bomb survivors tell their second-generation children about their experiences, and how the second-generation accepts these stories, would be a fruitful line of inquiry.

### Health Anxiety about Genetic Effects of Radiation Exposure

The results showed that, from childhood to adolescence, participants had not developed health anxiety about the genetic effects of radiation exposure; however, it was observed that, in some cases, participants developed health anxiety about themselves and their children once they reached adulthood. When the SGS did not have any physical problems, they did not have anxiety; however, health anxiety could be activated when triggered by physical illness.

It was possible to distinguish those who were certain that their illness was due to the effect of radiation exposure from those who did not. We believe that anxiety concerning genetic effects due to radiation exposure changes within one’s life according to the health status of the second-generation themselves and that of their children. From this, having no health anxiety at this time does not mean that anxiety will not occur in future, and that health anxiety may lie dormant within the atomic bomb survivors and their descendants. Just as anxiety and PTSD reactions increased among third-generation atomic bomb survivors after the Fukushima nuclear incident (Ben-Ezra et al., 2012), it is conceivable that SGS’ anxiety will increase after radiation-related incidents occur and after they enter middle age when the morbidity rates for cancer rise. In the future, there will likely be a need for psychological support for this anxiety.

In this study, some of the SGS developed health anxiety about their third-generation children, but only female participants. Several studies revealed vulnerability factors related to the psychological impact of the Chernobyl accident. Females and adults with children appear to be especially vulnerable (Havenaar et al., 1997; Foster and Goldstein, 2007). From these results, it can be said that the transgenerational transmission of anxiety concerning the genetic effects of radiation exposure causes specific stress to women with children.

Moreover, Kim et al. (2011) also observed that aspects of “persistent distress” in the atomic bomb survivors stemmed from a lack of knowledge regarding radiation effects and a fear of learning about their potential risk. However, the fact that many of the survey respondents felt anxious about their health, even though they knew that there was no medical support of the effects from radiation demonstrates, clearly illustrates that access to medical information does not necessarily mean that their anxiety is entirely dissipated. It is conceivable that atomic bomb survivors and their descendants are likely to feel anxiety in the form of uncertainty in the face of an unexplained menace that is not thoroughly validated scientifically. Meanwhile, among the second-generation respondents who were certain of genetic effects were those who did not themselves have any serious illnesses. This suggests the possibility that their health anxiety had causes other than their health status. It is necessary to study correlations between the family dynamic, including parent–child relationships; other personal history causes, intolerance of uncertainty (Buhr and Dugas, 2002), and other personality factors as causes of health anxiety in the second-generation.

### Limitations and Directions for Future Research

There are several limitations to this study. I employed a limited number of sample size partly recruited by a snowballing method, which may result in a selection bias. Future research needs to replicate the findings from this study, for example, employing quantitative methods.

All but one of the survey respondents in this study were born and raised in the bombed regions. The results of this study indicated the possibility that, for those born and raised in the bombed regions, the substantial number of atomic bomb survivors in the communities acted as a deterrent to discrimination and prejudice. As such, it would be useful to compare the psychological effect on the second-generation living outside of the bombed areas, including those living outside of the country.

This study was unable to examine the relationships between the survivors and the SGS, and how the survivors communicated their experiences to the second-generation. A worthwhile area of inquiry would be to study how trauma is transmitted intergenerationally, or, if the effect of trauma is attenuated in the second-generation, what kind of resilience contributed to that attenuation.

## Author Contributions

YK was involved in the study conception and design, acquisition of data, analysis and interpretation of data, drafting of manuscript, and the critical revision of the manuscript.

## Conflict of Interest Statement

The author declares that the research was conducted in the absence of any commercial or financial relationships that could be construed as a potential conflict of interest.

## Abbreviations

PTSD: post-traumatic stress disorder
SGS: second-generation survivors
